# Training of patient and consumer representatives in the basic competencies of evidence-based medicine: a feasibility study

**DOI:** 10.1186/1472-6920-10-16

**Published:** 2010-02-11

**Authors:** Bettina Berger, Anke Steckelberg, Gabriele Meyer, Jürgen Kasper, Ingrid Mühlhauser

**Affiliations:** 1 Unit of Health Sciences and Education, University of Hamburg, Martin-Luther-King Platz 6, 20146 Hamburg, Germany

## Abstract

**Background:**

Evidence-based medicine (EBM) has become standard approach in medicine. Patients and health authorities increasingly claim active patient roles in decision making. Education to cope with these roles might be useful. We investigated the feasibility, acceptability and possible impact of EBM training courses for patient and consumer representatives.

**Methods:**

We designed a generic one-week EBM course based on previous experience with EBM courses for non-medical health professionals. A course specific competence test has been developed and validated to measure EBM skills. Formative and summative evaluation of the course comprised: 1) EBM skills; 2) individual learning goals; 3) self-reported implementation after six months using semi-structured interviews; 4) group-based feedback by content analysis. EBM skills' achievement was compared to results gathered by a group of undergraduate University students of Health Sciences and Education who had attended a comparable EBM seminar.

**Results:**

Fourteen EBM courses were conducted including 161 participants without previous EBM training (n = 54 self-help group representatives, n = 64 professional counsellors, n = 36 patient advocates, n = 7 others); 71% had a higher education degree; all but five finished the course. Most participants stated personal learning goals explicitly related to practicing EBM such as acquisition of critical appraisal skills (n = 130) or research competencies (n = 67). They rated the respective relevance of the course on average with 80% (SD 4) on a visual analogue scale ranging from 0 to 100%.

Participants passed the competence test with a mean score of 14.7 (SD 3.0, n = 123) out of 19.5 points. The comparison group of students achieved a mean score of 14.4 (SD 3.3, n = 43). Group-based feedback revealed increases of self confidence, empowerment through EBM methodology and statistical literacy, and acquisition of new concepts of patient information and counselling. Implementation of EBM skills was reported by 84 of the 129 (65%) participants available for follow-up interviews. Barriers included lack of further support, limited possibilities to exchange experiences, and feeling discouraged by negative reactions of health professionals.

**Conclusions:**

Training in basic EBM competencies for selected patient and consumer representatives is feasible and accepted and may affect counselling and advocacy activities. Implementation of EBM skills needs support beyond the training course.

## Background

There are various reasons why patient and consumer representatives should be offered training in the basics of Evidence-based Medicine (EBM). First, EBM has become a standard approach to problem solving in medicine and health care [1]. Although EBM was originally designed for use by individual physicians to make decisions on medical problems of individual patients [2], the method has been adapted as a general approach to decision making in health care [1,3]. Second, patients and health authorities increasingly claim active patient roles in health care decision making [4,5]. Patients and consumers are already represented on health care boards, in agencies and institutions. They are members of ethical committees and are increasingly asked to take part in health technology assessment and patient information or guideline development processes applying EBM methodology [6]. Third, rationalising of medical decision making be it on an individual or on a public health care level is often poorly understood by patients. This leads to protests of disappointed patients who fear restrictions in health care supply [7,8]. Fourth, patients increasingly search the internet for up-to-date health information. Results of clinical trials are commonly promoted in a biased way by the pharmaceutical industry or allied medical experts and evidence based secondary (pre-appraised) sources remain limited [9]. Various patient groups already critically appraise research articles or provide summaries of clinical research on their websites. Patients could be better supported to make best advantage of their assessments.

Finally, patient participation in medical decision making requires provision of evidence-based patient information [10-12], empowerment of patients to use the information and counselling that considers concepts of informed and shared decision making [13,14]. Ethical guidelines demand evidence-based, clear and unbiased patient information and counselling in regard to diagnostic, therapeutic or preventive decisions [15]. However, adequate evidence-based patient information and counselling remains limited in practice [9]. One major barrier is the poor risk literacy among health care providers [16,17] and patients alike [17,18]. Patient and consumer representatives play a pivotal role in endorsing concepts of evidence-based patient information and decision making. As a prerequisite they need basic competencies to critically appraise and appropriately communicate scientific information [18], elements that are central to EBM [2].

Training courses in EBM are primarily offered for physicians and increasingly also for other health care professions [2,19,20]. At the time when this project was designed a systematic literature search identified only two initiatives to teach consumers or patient representatives basics of clinical science. In the United Kingdom Milne and Oliver had piloted a critical appraisal programme for people, who give health information to the public and members of maternity self help groups. The workshops involved 54 people, who attended pairs of half-day workshops. Outcome measures have been attendance at workshops, satisfaction and enjoyment of workshops [21]. In the United States the National Breast Cancer Coalition had developed special science training for breast cancer activists of the LEAD project (Leadership, Education, and Advocacy Development) [22]. LEAD graduates serve on influential research boards and committees in US federal and state governments, universities, hospitals and private industry [23]. Generic training programmes on EBM skills for consumer and patient representatives who were medical laypersons had not been reported.

We developed and piloted a one-week EBM training course for consumer and patient representatives. Graduates of the programme should achieve basic understanding of the relevance and uncertainty of science in health care and should be enabled to apply basic EBM skills that are relevant to patient and consumer representatives. We investigated the feasibility and acceptability and the potential impact on the implementation of basic EBM competencies.

## Methods

Our study covered three pre-defined phases: 1) Curriculum development, 2) development and validation of the competence test, and 3) pilot testing of the training courses.

### 1) Curriculum development

Based on the original concept of the five-day EBM courses for international participants held at the EBM centre of Oxford University in the late nineties [2] we designed and piloted EBM courses for non-medical students and health professionals without academic training [24,25]. Feasibility, acceptability and knowledge acquisition were demonstrated for these courses and we felt confident that it could be adapted for medical laypersons who act as patient or consumer representatives and professional counsellors. Specific needs of our target groups were elicited by screening available experience [21,22]. In addition, we performed two pilot courses involving 25 participants. The results from the two pilot courses indicated that only minor changes of the training course were necessary. We also tested a 2 × 3-day format including one month break between the two parts.

Underlying theories and concepts are presented in the following paragraphs. Additional file 1 displays an overview of the final structure, specific objectives, topics, materials, and methods of the EBM training courses.

### Didactic analysis

The courses were adapted for patient and consumer representatives using the theoretical framework of critical-constructive teaching methods of Klafki, which focuses on self-determination and participation abilities of students [26]. The theoretical model of Klafki promotes systematic reflection regarding aims and intentions of instructions as a prerequisite for the development process of a curriculum.

### Evidence based medicine as a problem solving strategy

We applied learning strategies developed for adult teaching [27]. We assumed that people would have a strong internal motivation if their personal experiences as patients or patient counsellors are met. Therefore, participants were given as much opportunity as possible to work and discuss in small groups in order to bring in their own experiences. Our teaching concept was based on cognitive learning and teaching principles [28] in which the teacher demonstrates methods to solve problems and enables participants to transfer these methods to their own fields. We used and adapted the core elements of EBM [2] which included the following steps: 1) ask a question that can be answered; 2) identify appropriate sources for searching relevant information and perform a systematic literature search; 3) critically appraise selected publications on key elements; 4) communicate study results to patients and consumers. Additional teaching sessions specifically targeted 1) the basics of statistics, 2) consumer information and the media, 3) risk communication, 4) clinical testing of new drugs and drug approval, and 5) the role of patient representatives in institutional review boards.

We used various topics of controversy in medicine and health care to demonstrate the relevance and the general principles of the method of EBM: Hormone therapy in (post-) menopausal women [29-31] was used to exemplify the fallacies resulting from reliance on epidemiological studies and surrogate parameters rather than on evidence by high quality controlled trials and patient relevant outcomes. Screening for colorectal cancer was used to teach quality criteria for diagnostic tests, evaluation of screening programmes and aspects of communication about benefits and risks of screening interventions [32]. A meta-analysis on the effects of homoeopathy [33] was critically appraised to exemplify the limitations and strengths of systematic reviews and meta-analyses. Two weeks prior to the commencement of the courses all participants received a handbook of study materials for advance preparation. The handbook comprised about 60 pages with publications, vocabularies, glossaries, work sheets and supplementary information.

### 2) Development and validation of the competence test

A systematic literature search identified several evaluation instruments for EBM programmes [34-38]. Since these instruments targeted courses for physicians and did not evaluate the skills required to communicate study results we judged them unsuitable for medical laypersons and patient representatives. Therefore, we developed a new questionnaire to assess knowledge and skills based on theoretic concepts and teaching materials developed for students and health care professionals. Five areas of evaluation reflecting the core competencies were defined: 1) "question formulation" including competencies in outline design, target population, intervention, control, and relevant outcome parameters of a clinical study (prevention of myocardial infarction by Vitamin E [39] was used as an example); 2) "literature search" including competency to define relevant search terms and to perform a search in the medical literature database PubMed; 3) "reading and understanding" including competency to identify study aim, number of participants, duration and location of the study, study and control interventions, and primary endpoints; 4) "calculation" including competency to calculate the event rates reported in controlled trials, the absolute and relative risks of getting a certain event, the risk reduction or the risk increase, caused by the intervention examined, and the number needed to treat or the number needed to harm using the 2 × 2 table; 5) "communication of study results" including competency to outline general aspects of evidence-based patient information and to express numbers in layperson terms as meaningful and understandable patient oriented statements. The questionnaire comprised 19 items. Possible scores ranged from 0 to 19.5. Answers were scored as 0, 0.5 or 1. Content validity was checked by an external expert in EBM who had not been involved in item construction. We pilot tested the questionnaire with four students at the University of Hamburg for wording and usability. Reliability and item properties of the competence test were determined within the two EBM pilot courses involving 25 participants.

To show validity of the competence test we investigated its sensitivity for EBM competency change in a group of undergraduate students of Health Sciences and Education. All students were non-medical health professionals before their University studies. Content and methods of the students' EBM course were comparable to the curriculum of the training for patient and consumer representatives. We asked the students to fill in the questionnaire before and after the EBM course. We considered a training effect of five score points as relevant. Sample size was calculated, intending a 90% power, accepting 5% alpha error and adjusting for a standard deviation of 5.9 score points. The latter value was taken from the piloting of the competence test. Based on these assumptions a group of 17 participants were required. Values were compared by paired t-test.

A total of 22 consecutive students completed the questionnaire before and after their participation in the EBM course. An additional group of 21 students participated in after course assessment only. Test results were rated by two independent researchers showing high interrater reliability (kappa 0.97). The mean change gathered by the 22 students was from 4.8 (SD 1.2) before to 13.5 (SD 3.7) scores after the course (p < 0.0001) indicating the validity of the instrument. The total after course sample of students (n = 43) reached a score of 14.4 (SD 3.3).

### 3) Pilot testing of the training courses

The target group of our EBM programme consisted of professional counsellors, members of self-help groups in Germany [40,41], and professional patient advocates. We invited persons who belonged to one of these groups and expressed willingness to develop skills to critically appraise scientific literature and to use their new competencies on behalf of patient interests.

Recruitment strategies comprised announcements through newsletters, mailing lists, flyers, newspaper publications and self-help networks. Participation was free of charge. The courses took place at the University of Hamburg. The programme was accredited by three German federal states as paid five-day educational leave enabling participants in full time state employment to join the course. Some participants used their annual leave to join the programme. We offered 10 courses as one week courses from Monday to Friday and four courses as 2 × 3 days from Thursday to Saturday.

### Evaluation

Formative and summative elements of evaluation were combined [42]. Formative evaluation was used to improve programme performance. Evaluation sheets on teaching quality and content of the course modules were distributed daily. Summative evaluation of the programme aimed to verify that participants 1) were able to understand and acquire the methods of EBM; 2) regarded the adoption of EBM methods as personal learning goal; 3) could transfer the methods into their own area; and 4) whether the subgroups (laypersons, mainly self-help group members, professional counsellors, and professional patient advocates) differ in educational background, learning goals and implementation of gained knowledge and skills.

We also performed a group-based evaluation. Perceived benefits and deficits of the course programme were discussed in groups and surveyed using Metaplan [43]. The content of the Metaplan cards was part of the summative evaluation and used for the content analysis [44].

### Acceptability

To assess acceptability we developed a purpose-based assessment instrument. We aimed to find out, if 1) participants were enthusiastic about adopting EBM methods; 2) our programme met the individual learning goals of the participants; 3) any subgroups differed in their evaluation of the programme. The baseline personal learning goals were assessed by telephone interviews two to three days before each of the courses, assigning the answers to the main categories of learning goals, identified during the pilot courses. Nine main categories were identified which turned out to be meaningful to participants: (1) "research skills", (2) "critical appraisal skills", (3) "communication skills", (4) "advanced education", (5) "understanding of EBM", (6) "networking", (7) "empowerment", (8) "implementation", (9) "others". These categories were used to assess acceptability. Participants were asked to evaluate every module of the main course related to their personal learning goals using visual analogue scales with a scope from 0 to 100 percent. Differences between target groups have been tested by unpaired t-test.

### EBM competencies

To estimate an increase in EBM competencies we used the validated competence test. Participants were informed about pseudonymised data analysis and given option to withdraw from the study at any time. The questionnaire was completed at the end of the course. We chose not to perform a before-after test since the questionnaire took about four hours to complete. Instead, we compared the test results with those of the University students in Health Sciences and Education (see above), who had completed the comparable training. We assumed an unpaired t-test to show no significant difference between these two groups.

### Evaluating long-term implementation

We assessed the long-term implementation of EBM skills using semi-structured telephone interviews six months following the course. We asked participants to comment on areas of successful implementation, barriers to implementation, and further needs to implement the acquired skills. Notes from the interviews were categorized into two types of implementation: 1) use of critical appraisal skills; 2) activation of participants to take part in health care decision making. The first type of implementation covers five different potential levels of implementation:

• Level 0 (no implementation): participant reported no practice of EBM skills;

• Level 1 (minor implementation): participant reported a change in attitude and limited attempt to critically evaluate patient information or expert based opinions;

• Level 2 (fair implementation): participant reported use of selected skills such as literature search, critical appraisal of patient information and scientific literature;

• Level 3 (implementation of major components): participant reported to have developed a question which could be answered by systematic literature search and had performed a literature search or critically appraised an original study;

• Level 4 (almost complete implementation): participant reported application of almost all elements of EBM methodology and had produced a patient information or teaching programme or developed teaching modules.

Telephone interviews six months after the intervention with participants of the two pilot courses were used to construct categories for content analysis [44]. In a first step, two raters independently generated categories. Disagreement was solved by discussion.

### Summative analysis of group-based feedback

Group-based feedback of all courses was analysed using qualitative content analysis methods [44].

## Results

### Participants

Between September 2002 and April 2005, 14 courses were conducted involving 161 participants from 84 German and three Austrian institutions. One third of the participants (n = 54) were active representatives of self-help groups, 40% (n = 64) were professional counsellors, 22% (n = 36) were professional patient advocates, and 4% (n = 7) did not belong to our target groups. A total of 114 (71%) participants had a higher education degree [40 (25%) had attended a University of Applied Sciences and 74 (46%) a University], eight (5%) participants had a PhD. The remaining 34 (21%) participants had vocational training; data on seven participants (4%) are missing. The majority of participants were females [n = 127 (79%)].

### Acceptability

Participants with the personal learning goals "research skills" (n = 67), "critical appraisal skills" (n = 130), "communication skills" (n = 81), "advanced education" (n = 31), or "understanding of EBM" (n = 40) (multiple answers were possible) rated the relevance of the whole course for their personal learning goals on average at 80% (SD 4) on a visual analogue scale ranging from 0 to 100%. Participants with the objectives "networking" (n = 25), "empowerment" (n = 18), "implementation" (n = 6), or "others" (n = 2) rated the relevance of the whole course lower [65% (SD 2)]. There was only a weak correlation between relevance for personal learning goals and subjective evaluation of teaching quality or content of the course modules (r = 0.41, n = 123). This means that only about 17% of variation in acceptability could be attributed to the rating of didactic and content of the course programme [45]. This finding can be interpreted as an indicator for judgement of relevance, independent from the teaching performance of the course units. There was no significant difference between the three main target groups regarding judgement of relevance of the whole course for their personal learning purposes.

### Summative analysis of group-based feedback

Summarising and analysing participants' written reports resulted in the following achievements:

(1) Higher self-confidence: Participants redefined their roles within the health care system and appreciated not having to rely only on the medical doctor as an expert, but to be able to identify and use other sources of information.

(2) Recognising the limits of clinical research: Participants discovered that there are many uncertainties surrounding clinical research results and, therefore, critical appraisal of publications is necessary.

(3) Empowerment through EBM methodology: Participants realised that EBM is a method for critical appraisal of scientific studies which can be used to a certain extent even by laypersons.

(4) Empowerment through statistical literacy: Initially, many participants were afraid of using numbers. They felt encouraged when they learned how to calculate and interpret event rates and the numbers needed to treat or harm.

(5) EBM as a method to reflect own actions: Participants became aware of bias in clinical research and of the danger to selectively use only study results which support own points of view.

(6) New concepts of patient information and counselling: Patients adopted their right to know benefit, harms, and lack of benefit of medical interventions. They felt prepared to make informed decisions about treatments and to counsel and support others.

The following limitations of the courses were mentioned: Language barriers due to the English language of original publications, limited access to publications, too little time for reflection and discussion of the course content, lack of interest of physicians and pharmaceutical industry in patients' personal responsibility, insufficient readiness of patients to overtake responsibility, feelings of resignation due to complexity.

### EBM-competencies

Of the 161 participants a total of 148 filled in the questionnaires, eight declined to participate, and five did not finish the course programme. Data of 25 participants of the two pilot courses were excluded from analysis because they had already been used for test validation. The 123 participants who had completed the final version of the questionnaire achieved a mean score of 14.7 points (SD 3.0) out of a maximum score of 19.5. Differences between the scores of study participants and students were not statistically significant. Results are summarised in Table 1.

**Table 1:** Results of the competence test

	EBM related knowledge and skills (maximum score 19.5)
**Participants at the end of the course**	
Self-help group representatives (n = 43)	13.6 ± 3.1
Professional counsellors (n = 45)	15.2 ± 3.0
Patient advocates (n = 29)	15.8 ± 2.5
Others (n = 6)	13.4 ± 2.7
Total (n = 123)*	14.7 ± 3.0
**Undergraduate University students**	
Before training (n = 22)	4.8 ± 1.2
After training (n = 22)	13.5 ± 3.7
After training (n = 43)	14.4 ± 3.3

Values are means ± standard deviation

* Data of n = 38 participants were not included as results of n = 25 have been used for validation of the questionnaire, n = 8 declined to fill in the questionnaire, and n = 5 did not finish the EBM course.

### Long-term implementation

Out of the 156 participants who had finished the training course a total of 129 were available for follow-up evaluation by telephone interview. Participants used the acquired skills on different levels and in various ways, depending on previous experience and institutional background (Table 2). Self-help group members reported the most successful use of the course programme: 15 participants out of 54 (28%) felt encouraged to take up advocacy activities and 10 (18%) stated implementation, which we ranked high (Level 4). Professional counsellors predominately stated implementation at Level 2, whereas professional patient advocates stated implementation at Level 2, Level 3, and Level 4 at comparable degrees. However, 45 of the 129 interviewed participants (35%) reported not to practice any EBM skills.

**Table 2:** Levels of implementation of EBM-skills and advocacy uptake

	Self-help group representatives (n = 54)	Professional counsellors (n = 64)	Patient advocates (n = 36)	Others (n = 7)	Total (n = 161)	
Lost to follow-up	10	11	5	1	27	(17)
Did not finish the training course	2	0	3	0	5	(3)
**Follow-up interview**	**42**	**53**	**28**	**6**	**129**	**(80)**
Implementation*						
Level 0	16	18	8	3	45	(35)
Level 1	4	3	2	1	10	(8)
Level 2	7	22	8	2	39	(30)
Level 3	5	4	5	0	14	(11)
Level 4	10	6	5	0	21	(16)
Additional uptake of advocacy activities	15	9	6	0	30	(23)

Values are numbers (percentage)

* For explanation of levels of implementation see Methods and Additional file 2. Higher levels indicate more implementation.

Structured interviews revealed various barriers to implementation. Professional counsellors stated they had limited time to undertake systematic search and critical appraisal. They complained about a lack of evidence based patient information and decision aids, and the lack of quality standards in counselling. Members of self-help groups reported lacking opportunities to use EBM skills. They also complained about a lack of further support and possibilities to exchange experiences beyond the programme. Some members (n = 15) mentioned restrictions due to personal bad health condition. Several self-help group members felt discouraged to use their new skills because they had experienced negative reactions from professionals if they raised critical questions concerning therapeutic issues. Nearly a quarter of the participants expressed the need for long-term support related to counselling and communication training or further education. Examples of successful implementation are provided in Additional file 2.

## Discussion

The present study suggests that training of selected patient and consumer representatives in the basic competencies of EBM is feasible and accepted. Participants gained EBM skills comparable to those of undergraduate students of Health Sciences and Education.

Self-reported practice of EBM elements disclosed a diversity of use and a wide range of intensity of use. Comparable phenomena have been observed for EBM courses targeting health professionals [19,23,24]. At the highest level of implementation and achievement consumer representatives and patient advocates used skills to develop or revise own training programmes such as the courses for consumers within the Haematological Malignancies Cochrane Group in Cologne, Germany [46]. Participants of a German breast cancer advocacy group organised annual five-day courses and included elements of the EBM training such as critical appraisal of scientific publications and risk communication. As a result of the EBM training they submitted a petition to the German national parliament demanding continuous up-to-date information about clinical trial publications, putting EBM decision making into practice, and guidelines which explicitly have to be evidence-based [47].

### Barriers to implementation

Most barriers reported by study participants have also been reported for other EBM courses [19,23,24] such as difficulties in reading publications in English, organisational problems including limited access to databases and publications, time constraints or lack of further support and possibilities to exchange experiences beyond the programme.

### Strengths of the present study

Our study has some strength. This is the first programme teaching EBM skills to self-help group members and consumer representatives with a general rather than disease-specific focus. Our course allowed us to train a heterogeneous group of medical laypersons. More than 80 different institutions and organisations were represented by the study participants.

### Limitations of the present study

Our study has several limitations. In this pilot study we did not compare the training courses with a control group without intervention or a different kind of training. In addition, study participants were of higher education and particularly motivated as they expressed willingness to develop skills to critically appraise scientific literature (in English) and to use their new competencies on behalf of patient interests. Furthermore, through their personal careers and particular roles they were not entirely laypersons in medicine. Offering such programmes or parts of it to other patients or consumers would need further simplification and adaptation.

The long-term evaluation of the study is only descriptive and assessors were not blinded. Therefore, it is not possible to differentiate or quantify intervention-related effects.

The curriculum needs updating. EBM is an ever developing method. Since we have designed the project in 2001 searching of databases has improved and secondary sources with pre-appraised information are increasingly offered. Finally, the long duration for performing the competency test limits its practicability. Therefore, we have developed a new instrument for evaluation of EBM skills [48].

## Conclusions

Training in the basic competencies of EBM for selected and motivated patient and consumer representatives is feasible and accepted and may impact on counselling and advocacy activities. The study had major impact on the development of similar trainings like the ones of the Women's Health centre in Graz, Austria [49] and also on the ebm@school curriculum for secondary school students [50].

## Competing interests

The study was financed by the German statutory health insurance system (GKV) within the framework of a national model project for patient and consumer information centres according to §65b of the German Social Security Code V.

The authors declare that they have no competing financial interests. However, all authors are strongly dedicated to empowerment of patients and consumers which might be considered to constitute an intellectual conflict of interest related to this manuscript.

## Authors' contributions

All authors have made substantial contributions to conception and design of the project. IM, GM, AS and BB developed the curriculum and performed the courses. BB designed the questionnaires, developed categories for long-term implementation, interpreted the data and drafted the paper. IM acted as EBM expert for the content validation of the competence test. JK gave methodological support over the whole study period. GM, AS and IM commented on paper drafts. All authors read and approved the final manuscript.

## Authors' information

BB has been active in patient advocacy for many years. She has studied intercultural sciences and has a PhD in Health Sciences and Education. Her main research area is qualitative research in patient empowerment and development of training courses for informed decision making. She works at the Unit of Medical Theory and Complementary Medicine, University of Witten/Herdecke, Gerhard-Kienle-Weg 4, 58313 Herdecke, Germany.

AS has vocational training as a nurse, has studied Social Sciences and Health Sciences and has a PhD in Health Sciences and Education. Her main research area is evidence-based information of patients and the public and development of training courses in health literacy for school students.

GM has vocational training as a nurse, has studied German Language and Health Sciences and has a PhD in Health Sciences and Education. At present she is professor for clinical nursing research at the Institute of Nursing Science, Faculty of Medicine, Witten/Herdecke University, Stockumer Straße 12, 58453 Witten, Germany.

JK has a PhD in Psychology. His research areas are communication between patients and physicians with a focus on development of evidence-based patient information and shared decision making.

IM is a medical doctor of internal medicine, diabetologist and endocrinologist. She holds a professorship of Health Sciences and Education. Research areas are structured patient education, evidence-based medicine, patient information and informed decision making in health care.

## Pre-publication history

The pre-publication history for this paper can be accessed here:

http://www.biomedcentral.com/1472-6920/10/16/prepub

## Supplementary Material

Additional file 1 **Curriculum**. Structure, specific objectives, topics, materials, and methods of the evidence-based medicine (EBM) training courses Click here for file

Additional file 2 **Examples of implementation**. Examples of successful implementation as reported by participants Click here for file
