# Supplementary material for: Training of patient and consumer representatives in the basic competencies of evidence-based medicine: a feasibility study
Source: BMC Med Educ. 2010 Feb 11;10:16. doi: 10.1186/1472-6920-10-16 (PMC2843725; doi:10.1186/1472-6920-10-16)
Supplement: Additional file 1 — Curriculum. Structure, specific objectives, topics, materials, and methods of the evidence-based medicine (EBM) training courses [file 1472-6920-10-16-S1.DOC]

### Additional file 1 – Structure, specific objectives, topics, materials, and methods of the evidence-based medicine (EBM) training courses

| Time  min | Objectives:  Participants should … | Topics | Materials and methods |
| --- | --- | --- | --- |
| 30’ | - differentiate between expert-based and evidence-based information - know examples of fallacies of medical / health issues | Reasons for training laypersons in EBM methodology and skills | Introduction into EBM.  Examples of expert-based treatment fallacies with fatal and non-fatal consequences. (Lecture, discussion, worksheets, flip charts) |
| 180’ | - define methodological and statistical terms like sample size calculation (α and ß error) - know the relevance of a clinically important question and statistical hypothesis - know the differences between fishing for p-values versus predefined outcomes | Basics of statistics | Worksheets, simulation of statistical issues by gambling. (Lectures, exercises) |
| 45’ | - understand and reflect how expert information on treatment benefit and safety are presented in public media | Information of consumers through public media | Sections from two video-taped TV features on hormone replacement therapy; worksheet with key questions.  (Observation and plenary discussion) |
| 180’ | - understand which evidence is needed to draw conclusions about efficacy and safety of an intervention | Fallacies of observational research | Abstract and Tables of the Nurses Health Study [1], English-German vocabulary list and critical appraisal sheet. (Group work, plenary discussion, computing) |
|  | - understand the structure of a scientific paper - select basic information and recognize bias and potential confounders - calculate relative risk reduction |  | Displaying a fictitious observational study on associations between short skirts and beautiful legs. (Presentation and plenary discussion) |
| 90’ | - define questions relevant to patients and consumers - draft a research question | Developing a question, which could be answered by systematic literature search | Flip chart; lecture; worksheets; mind mapping. (Presentation and individual work) |
| 90’ | - perform a PubMed database search (http://www.pubmed.gov) - apply operators (AND, OR, NOT, NEAR) - apply limits, truncations, thesaurus and free text - access or order an original study | Introduction to databases,  systematic literature search | Handouts comprising general information on biomedical databases and relevant internet addresses. (Individual work at computers) |
| 90’ | - reconstruct study designs to generate evidence regarding the effectiveness of interventions | Randomized controlled trial (RCT) | Fictitious story about a person with skin problem; development of an experimental study design. Computing benefit and lack of benefit of a fictitious treatment for a skin problem compared to placebo. (Presentation and plenary discussion) |
|  | - understand study flow, baseline data, and results of the Women's Health Initiative on primary prevention of cardiovascular disease through hormone replacement therapy [2] |  | Abstract and Tables of the Women's Health Initiative [2]; English-German vocabulary list and critical appraisal sheet. (Group work, plenary discussion, computing, lectures) |
| 90’ | - understand strategies of framing of data - understand relative risk reduction / increase (RRR), absolute risk reduction / increase (ARR) and number needed to treat / harm (NNT / NNH) | Framing of data | Computing event rates, absolute and relative risk, risk reductions, and number needed to treat by 2x2 tables. (Presentation and individual work using calculators) |
|  | - critically appraise patient information |  | Misleading patient information sheet on hormone replacement therapy distributed by gynaecologists in private practices. (Individual work and plenary discussion) |
| 180’ | - know EBM as a suitable method for various study questions | Critical appraisal of systematic reviews and the role of the Cochrane Collaboration | Worksheet on pros and cons to use EBM methods to investigate complementary and alternative medicine. (Plenary discussion) |
|  | - know the Cochrane Collaboration as producer of systematic reviews on various topics in health care |  | Introduction to the Cochrane Collaboration. (Lecture) |
|  | - understand methods and aims of systematic reviews - reflect the publication bias - apply criteria of critical appraisal of systematic reviews |  | Systematic Review on homoeopathy [3], English-German vocabulary list, and critical appraisal sheet. (Small group and plenary discussion) |
|  | - search for and access systematic reviews |  | Internet access to the Cochrane Library.  (Individual work) |
| 90’ | - understand possible test results (positive / false positive, negative / false negative) - explain quality criteria of diagnostic tests (sensitivity, specificity, positive and negative predictive values) - understand the impact of prevalence on predictive values - define precision and accuracy of diagnostic tests | Diagnostic tests and screening interventions | Worksheet comprising items on validity of diagnostic tests. (Work in pairs and plenary discussion)  Abstract and tables of an RCT on screening for colorectal cancer [4]; English-German vocabulary list and critical appraisal sheet |
|  | - know the ethical implications of screening interventions - know benefit and lack of benefit and harm of screening - know framing of data in information on diagnostic tests | Ethical guidelines of screening information | Worksheet presenting ethical considerations on consumer information about screening programmes. (Work sheets, discussion, lecture) |
| 90’ | - use study results to inform patients - present study results using criteria of evidence-based patient information | Communicating interventions' benefit and lack of benefit to consumers and patients | Worksheet comprising questions on balanced reporting of benefit, lack of benefit, and adverse effects of interventions.  (Paperboard for working groups. Plenary presentation of results) |
| 180’ | - know basic procedures of drug approval and the relevance of institutional review boards (IRB) - know the opportunities and responsibilities of patient representatives in IRBs - critically appraise information sheets for study participants | From clinical testing of new drugs to drug approval  and the role of patient representatives in IRBs | Overview about the drug approval, examples of information for study participants (accepted by IRB) and checklists to appraise consumers' information.  (Group works and plenary discussion) |

1. Grodstein F, Stampfer MJ, Manson JE, Colditz GA, Willett WC, Rosner B, Speizer FE, Hennekens CH: **Postmenopausal estrogen and progestin use and the risk of cardiovascular disease.** *N Engl J Med* 1996, **335:**453-461.

2. Writing Group for the Women's Health Initiative: **Risks and benefits of estrogen plus progestin in healthy postmenopausal women: principal results from the women's health initiative randomized controlled trial.** *JAMA* 2002, **288:**321-333.

3. Linde K, Clausius N, Ramirez G, Melchart D, Eitel F, Hedges LV, Jonas WB: **Are the clinical effects of homeopathy placebo effects? A meta-analysis of placebo-controlled trials.** *Lancet* 1997, **350:**834-843.

4. Kronborg O, Fenger C, Olsen J, Jorgensen OD, Sondergaard O: **Randomised study of screening for colorectal cancer with faecal-occult-blood test.** *Lancet* 1996, **348:**1467-1471.
