# Supplementary material for: Training of patient and consumer representatives in the basic competencies of evidence-based medicine: a feasibility study
Source: BMC Med Educ. 2010 Feb 11;10:16. doi: 10.1186/1472-6920-10-16 (PMC2843725; doi:10.1186/1472-6920-10-16)
Supplement: Additional file 2 — Examples of implementation. Examples of successful implementation as reported by participants [file 1472-6920-10-16-S2.DOC]

**Additional file 2 – Examples of implementation**

**Level 1 – Minor implementation**

Using EbM knowledge to communicate with clients

- asking for evidence-based patient information

Using EbM knowledge to communicate with experts

- posing critical questions in public lectures about trial methodology
- discussion of the problem of publication bias in public

**Level 2 – Fair implementation**

Systematic literature search for original studies

- on topics like “long term outcomes of surgery for sclerosis”, “physiotherapy in rheumatic arthritis”, “nutritional supplements in prostate cancer”, “lung cancer and chemotherapy”, “Yoga”
- members of self-help groups with rare diseases noticed the lack of studies on therapies of rare diseases
- more careful counselling on preventive options

**Level 3 – Good implementation of major components of EbM-skills**

Critical appraisal of original literature

- research about self management programmes for patients with bipolar mania and decision to become an active member of a guideline group
- research about “sudden child death”

Development of a critical attitude

- Initiation of a working group dedicated to EbM in rare diseases

**Level 4 – Almost perfect implementation**

Development of patient information material

- on prenatal diagnosis and sudden child death
- on Multiple Sclerosis (MS) within a journal club: participants with MS met every month to critically appraise studies on MS therapy and diagnosis, results are published online (www.gesundheit.uni-hamburg.de)

Reflection of professional activities

- e. g. training programmes on breast self examination

Development of own training programmes or teaching modules

- training programmes for consumers offered by the Cochrane Haematological Malignancies Group, Cologne

**Uptake of advocacy activities in addition to implementation of EbM-skills**

- starting to work as patient representative
- starting to work in an institutional review board (IRB), in a guideline group, and in a research committee
- starting activity as patient representative in the German Federal Joint Committee for health issues
- stop working as IRB member due to increased awareness about the responsibility of IRBs
